# Inferior mesenteric plexus block under computed tomography guidance: A case report

**DOI:** 10.1097/MD.0000000000025866

**Published:** 2021-05-14

**Authors:** Jun-Mo Park, Seong-Min Hwang

**Affiliations:** a Department of Anesthesiology and Pain Medicine, School of Medicine, Kyungpook National University; b Department of Anesthesiology and Pain Medicine, Kyungpook National University Hospital, Daegu, South Korea.

**Keywords:** abdominal pain, autonomic nerve block, cancer, inferior mesenteric plexus, neurolysis, sympathectomy

## Abstract

**Rationale:**

Inferior mesenteric plexus block is indicated for left-sided lower abdominal pain. However, in patients with terminal cancer, severe abdominal pain can prevent the patient from maintaining the necessary posture during the procedure, and considerable anatomic deformation owing to extensive growth, invasion, and metastasis of the tumor in the abdominal cavity can make the procedure difficult. In these cases, performing the procedures under computed tomography (CT) guidance can ensure greater safety and accuracy.

**Patient concerns:**

A 63-year-old man was referred for severe left-sided lower abdominal pain. He was unable to lie prone owing to severe lower abdominal pain and right hip surgery performed 15 years ago. His visual analog scale score was 9 out of 10.

**Diagnoses:**

The patient had terminal pancreatic tail cancer. Compared with abdominal CT findings obtained 50 days ago, hepatic metastasis and peritoneal seeding were still present, infiltration to the tissues around the pancreas and retrogastric area was increased, and most of the abdominal aorta was encased. In addition, metastatic lymph nodes were identified in several areas on the left including the left para-aortic area. However, the lesion causing the pain could not be identified. Therefore, an inferior mesenteric plexus block was performed according to the patient's complaint.

**Interventions:**

Epidural patient-controlled analgesia was performed first. The patient's pain consequently reduced to a certain level, and the prone position became possible to some extent, so a CT-guided inferior mesenteric plexus block was performed 2 days later.

**Outcomes:**

After the CT-guided inferior mesenteric plexus block, it became possible to control the patient's pain with a fentanyl patch 75 mcg/hour only, and his visual analog scale score was reduced to 4. After 4 weeks, the patient died without complaints of severe pain as before.

**Lessons:**

CT-guided inferior mesenteric plexus block can be performed in patients with left-sided lower abdominal pain, enabling a safer and more accurate procedure especially in patients with terminal cancer who are unable to lie prone owing to severe lower abdominal pain or with considerable anatomic deformation due to extensive growth, invasion, and metastasis of the tumor in the abdominal cavity.

## 1 Introduction

The inferior mesenteric ganglion is located mainly around the origin of the inferior mesenteric artery and is distributed by forming a plexus along the course of the arterial branches.^[1]^ The inferior mesenteric plexus belongs to the aortic plexus, which is located in front of the abdominal aorta and is responsible for the sympathetic innervation of mesenteric, pelvic, and urogenital organs.^[2,3]^ It is supplied directly by the left L2 lumbar splanchnic nerve. Unilateral or bilateral lower abdominal pain is the specific indication for an inferior mesenteric plexus block or chemical neurolysis in patients with chronic diseases or cancer.^[4–7]^ However, the safety of the procedure is compromised when the patient is unable to execute a position suitable for the procedure owing to severe abdominal pain.^[8]^ In addition, when considerable anatomic deformation occurs owing to extensive growth, invasion, and metastasis of the tumor in the patient's abdominal cavity, the procedure may not be performed accurately and the possibility of side effects of the procedure can be increased.^[9]^

In this case report, pain control was implemented safely and accurately through a computed tomography (CT)-guided inferior mesenteric plexus block after epidural patient-controlled analgesia (PCA) in a patient with terminal pancreatic tail cancer in whom performing the procedure safely and accurately was difficult owing to severe abdominal pain and considerable anatomic deformation in the abdominal cavity.

## 2 Case report

A 63-year-old man was hospitalized for poor general condition accompanied by intensifying constipation and was referred for treatment of severe lower abdominal pain. His visual analog scale score was 9 out of 10. He had terminal pancreatic tail cancer, and comorbidities included diabetes mellitus, bronchial asthma, and hyperlipidemia. It was difficult for him to lie prone because of a surgery on the right hip that he had undergone 15 years ago. Unlike most patients with pancreatic cancer, the patient complained of lower abdomen pain, especially on the left side. He described his pain as achy and crampy and diffusely located in his left lower abdomen. The abdomen was distended slightly, and bowel sounds were significantly decreased. He was taking a lot of narcotic analgesics, which made him drowsy and lethargic and worsened his constipation. Simple abdomen X-ray imaging showed gas and abundant feces in the colon loops. Compared with abdominal CT findings obtained 50 days prior, hepatic metastasis and peritoneal seeding were still present, infiltration to the tissues around the pancreas and retrogastric area was increased, and most of the abdominal aorta was encased. In addition, metastatic lymph nodes were identified in several areas on the left including the left para-aortic area. However, on CT images, we could not identify the lesion that caused the pain. Nevertheless, owing to the patient's condition, we had to find a way to reduce the pain while reducing the dose of narcotic analgesics. Therefore, according to the patient's complaints, we decided to administer an inferior mesenteric plexus block instead of a celiac plexus block. However, the patient could not lie in the prone position at all.

At the outset, it was important to reduce the patient's lower abdominal pain by first performing epidural PCA so that the prone position was possible. Epidural PCA was performed under fluoroscopy guidance in the left lateral recumbent position. Fortunately, the patient's pain somehow decreased after epidural PCA, but epidural PCA alone did not control the patient's pain to a tolerable level. After 2 days of epidural PCA, it was still impossible for the patient to lie prone completely, so we proceeded with the procedure by using a cushion and blanket to make the patient feel less uncomfortable during the procedure. For safety, we started the procedure after confirming that the patient's posture would be maintained during the procedure. In addition, because of the considerable anatomic deformation owing to the extensive growth, invasion, and metastasis of the tumor in the abdominal cavity, we proceeded with CT guidance, instead of fluoroscopy.

**Figure 1:**
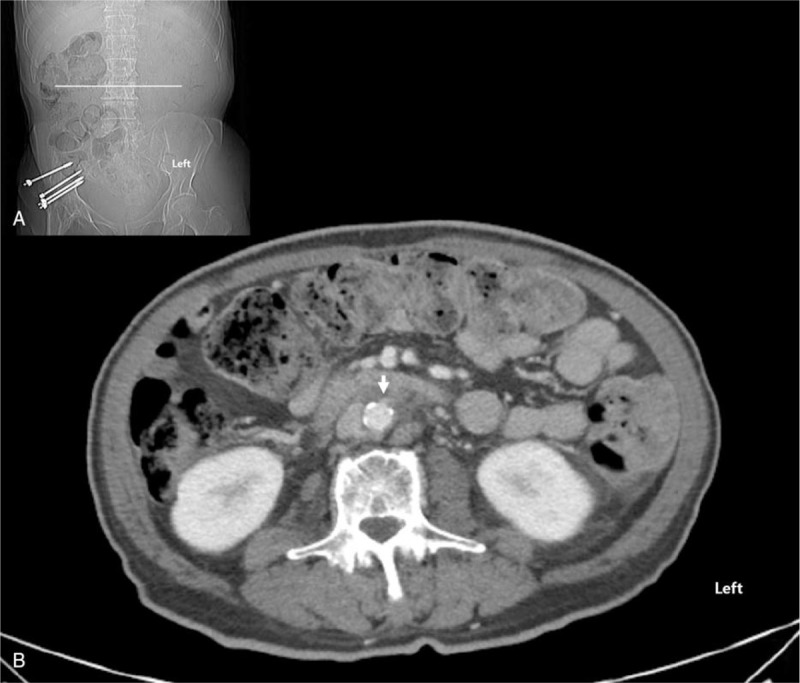
(A) Computed tomography (CT) scout view. (B) Axial CT image shows the inferior mesenteric artery (arrow) at L3 level.

**Figure 2:**
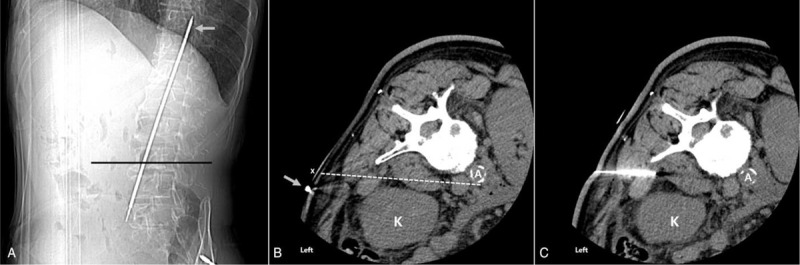
(A) Computed tomography (CT) scout view. (B) CT fluoroscopic image showing the skin entry point (X) and a safest and shortest imaginary line (dotted line) to reach the target point by avoiding the kidney. (C) CT fluoroscopic image showing the Chiba needle. Metal wire (gray arrow) and A (aorta).

**Figure 3:**
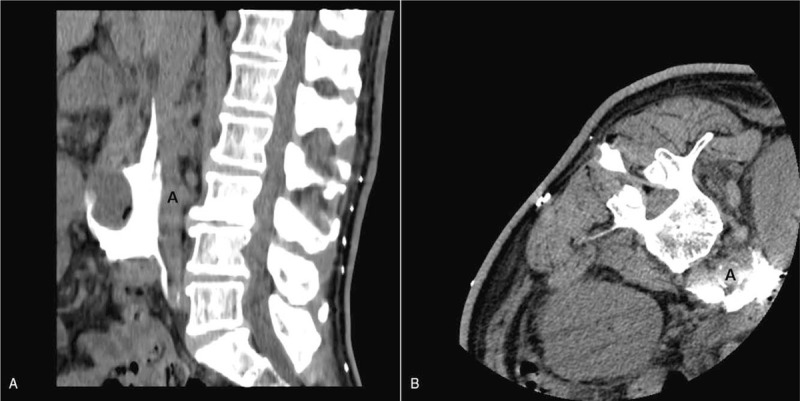
(A) Sagittal computed tomography (CT) image showing that the radiocontrast spreads well from the L2 to the L4 level in the front part of the abdominal aorta. (B) Axial CT image showing that the radiocontrast spreads well over the entire front part of the abdominal aorta. A (aorta).

Prior to the procedure, enhanced abdominal CT confirmed that the target site, inferior mesenteric artery, was located at the L3 level (Fig. 1). To find the most optimal skin entry point for the procedure under monitored anesthesia care, a metal wire was attached to the back, and CT was performed from the midportion of the L1 vertebral body to the midportion of the L5 vertebral body (Fig. 2A). On the axial CT fluoroscopy image, the skin entry point was selected, and a virtual line was drawn to the inferior mesenteric artery, which can be reached safely by avoiding the kidney (Fig. 2B). The skin entry point was marked at approximately 2 cm away from the metal wire toward the midline of the body, and the skin was sterilized aseptically and anesthetized using 2% lidocaine. Under the guidance of an intermittent axial CT fluoroscopy image, a 15-cm-long 22-gauge Chiba needle was slowly advanced toward the target point (Fig. 2C). After confirming that the Chiba needle arrived at the target point, 1 mL of radiocontrast was slowly injected, and CT scan was then performed to confirm that the needle tip was not located in the blood vessel. After an additional 3 mL of radiocontrast was slowly injected, CT scan was performed again to confirm whether the radiocontrast completely covers the area around the inferior mesenteric artery. If chemical neurolysis was required, we pre-emptively used 10 mL of 1.0% lidocaine to check whether the motor block was possible and 10 mg of triamcinolone to prevent a sudden increase in blood glucose in this patient with diabetes. The mixture of these 2 agents was slowly injected while checking the patient's response. After the administration of all the necessary drugs, CT scan was performed again to check the extent of the drug spread. The extent of the drug spread is shown in Figure 3. The patient was moved to the recovery room, where he was observed for 2 hours. There were no abnormal findings; therefore, the patient was sent back to the hospital room and the procedure was completed.

The patient did not show any side effects, and his visual analog scale score was reduced to 4. After the procedure, the patient's pain became tolerable using the fentanyl patch 75 mcg/hour only, except for the intermittent use of intravenous narcotic analgesics to control breakthrough pain. If the patient's pain increased and became worse within a few days, chemical neurolysis with alcohol was planned, but the patient did not complain of the same extreme pain as before for 1 week after the procedure. Both the patient and his family wanted to go to a nursing hospital near their house, which was more convenient for patient care. Because the patient's lifespan was not expected to be long, we only explained that neurolysis might be necessary, but did not implement it, and sent the patient to the desired hospital. The patient died after 3 weeks without complaints of severe pain as before.

Approval of this study was waived from the Ethics Committee of Kyungpook National University Chilgok Hospital, based upon their policy on case reports. The authors obtained written consent from the patient to publish this case report.

## 3 Discussion

In front of the infrarenal abdominal aorta between the left renal vein and the bifurcation of the common iliac arteries, several sympathetic ganglions are connected to each other by forming a network of parasympathetic and sympathetic nerves – an aortic plexus.^[1–3,10]^ Aortic plexuses include the celiac, aorticorenal, renal, superior mesenteric, intermesenteric, and inferior mesenteric plexuses. Nerves are distributed to the abdominal organs from these perivascular plexuses. Visceral afferent fibers carry pain information and travel with sympathetic fibers back to the spinal cord. Among them, the inferior mesenteric plexus and associated ganglia surround the inferior mesenteric artery. The sympathetic and parasympathetic contribution to the inferior mesenteric plexus is primarily from the lumbar splanchnic nerves (L2) and pelvic splanchnic nerves (S2–S4), respectively.^[11]^

Most patients with pancreatic cancer commonly complain of upper abdominal pain at the time of diagnosis and back pain even when it has progressed considerably.^[12]^ Herein, the patient was also diagnosed with pancreatic cancer because of epigastric pain 3 months before the referral. It is uncommon that a patient with pancreatic cancer complains of lower abdominal pain; however, as pancreatic cancer progresses, lower abdominal pain is possible with extensive growth, invasion, and metastasis of the tumor in the abdominal cavity. In addition, such pain is caused by the resulting ileus and severe constipation due to the use of narcotic analgesics. Even in patients receiving palliative care who are expected to have a short lifespan, as in this case, it is essential to consider in the differential diagnosis as to whether surgical treatment is essential. Amigo et al reported on the surgical treatment of a patient with gangrenous appendicitis whose symptoms were less severe due to the use of narcotic analgesics and corticosteroids while receiving palliative care.^[13]^ However, in this case report, the patient did not suddenly complain of lower abdominal pain, and abdominal CT performed 5 days before the referral did not reveal any lesions for which surgical treatment was deemed necessary.

In most pancreatic cancers, although a celiac plexus or splanchnic nerve block and chemical neurolysis is performed to achieve non-pharmacological pain control,^[12,14–16]^ they are mainly used for complaints of upper abdominal pain. However, abdominal CT confirmed that the area around the celiac trunk and superior mesenteric artery was severely encased, so it was very difficult to accurately implement celiac plexus block. Moreover, the patient's complaint of left lower abdominal pain was not an indication for celiac plexus block. It was regrettable that the exact cause of pain could not be identified on abdominal CT during the treatment course of this patient. In addition, 2 experienced radiologists confirmed that the recent abdominal CT did not find specific lesions that could be considered the cause of left lower abdominal pain. Therefore, as the patient complained of left lower abdominal pain, unlike most patients with pancreatic cancer, inferior mesenteric plexus block was performed. The inferior mesenteric plexus block is known to be useful for lower abdominal pain, especially for that occurring in the left side.^[4–7]^ As in this case, considerable anatomic deformation occurs if there are extensive growth, invasion, and metastasis of the tumor in the abdomen and the abdominal aorta and its major arterial branches are encased. This greatly hinders the safe and accurate implementation of the inferior mesenteric plexus block. Because of this, when the drug is injected into the patient, it is very likely that it does not spread accurately to the desired area. If the procedure is performed under fluoroscopy guidance as usual, it is nearly impossible to know exactly where the drug has spread. De Cicco et al reported that in patients with cancer or therapy-related regional anatomic distortion in the celiac region, neurolytic spread in the celiac region was highly hampered by regional anatomic alterations when a neurolytic celiac plexus block was performed.^[17]^

Inferior mesenteric plexus block has not been well described. Moreover, the difficulty and risk of the procedure is evident in patients with considerable anatomic deformation, as in this case. Although the CT-guided inferior mesenteric plexus block has a risk of radiation exposure, it has many advantages over fluoroscopy-guided procedure.^[18,19]^ CT has high contrast and spatial resolution and can clearly depict retroperitoneal structures and extent of tumor, and the exact location of the target site, inferior mesenteric artery, can also be checked. The location of the needle tip and surrounding structures can be visualized clearly, so an accurate procedure can be performed. CT fluoroscopy allows real-time monitoring of the procedure. Even if the exact location of the target site cannot be identified for various reasons, if the exact location of the target site is required, intravenous contrast agent can be used to define the exact location of the inferior mesenteric artery even during the procedure. CT can accurately predict the extent of diffusion of injected agents through radiocontrast injection and can check whether injected agents can leak into the peritoneal cavity. If these advantages are realized well, as in this case, a CT-guided inferior mesenteric plexus block makes the procedure much safer and more accurate.

Patients with terminal cancer are commonly unable to assume a posture suitable for the procedure owing to severe abdominal pain. In this case, the right hip surgery performed 15 years ago made it impossible for the patient to take a prone position. Thus, patients may find it difficult to maintain the prone position; consequently, the patient may suddenly move in the middle of the procedure because of pain or severe discomfort caused by the posture itself. Such an adverse event may have serious consequences. Although a significant number of patients have these difficulties, most of them tend to only receive drug treatment. In particular, patients with terminal cancer who are receiving palliative care often die without receiving any intervention that can provide considerable help other than receiving medications. In such cases, many people die while experiencing severe pain or side effects of narcotic analgesics. Fortunately, the authors confirmed that the patient could lie on his side, making epidural PCA possible, which could partially control the patient's pain. Through this, the patient was able to maintain a posture suitable for performing mesenteric plexus blockage under CT guidance.

Since our patient had terminal cancer and received palliative care, we tried to follow the practice guidelines for cancer pain management as much as possible.^[20]^ After inferior mesenteric plexus block, it was the authors’ understanding that chemical neurolysis using alcohol would not be performed if the patient was able to tolerate pain for the rest of his life with conventional medication alone. Fortunately, the patient lived for approximately 4 weeks after the procedure, but during that time, there was no complaint of extreme pain as before. If the patient had to undergo chemical neurolysis, the injection time would have been much longer to reduce the likelihood of serious side effects from alcohol or phenol, which would have put a considerable burden on the patient as he had to remain in the same position for a longer time. Chemical neurolysis is futile if the patient has a relatively short lifespan. Indeed, if severe pain continues to exist after the procedure, chemical neurolysis should be performed as the patient's pain would be relatively less to reduce the difficulty and burden of the procedure. Ideally, this would allow the patient to partially recover and die in severe pain-free conditions even without further intervention, which may bring about various risks and burden.

In conclusion, patients with terminal cancer may find it difficult to maintain a suitable posture for the procedure because of severe abdominal pain, so performing a safe procedure becomes challenging. In many cases, a procedure can be barely performed owing to considerable anatomic deformation due to the extensive growth, invasion, and metastasis of the tumor in the abdominal cavity. In this case, especially in patients complaining of left lower abdominal pain, inferior mesenteric plexus block and chemical neurolysis under CT guidance can be employed to ensure the safety and accuracy of the treatment procedures, thereby controlling the patient's pain.

## Author contributions

**Conceptualization:** Jun-Mo Park.

**Data curation:** Seong-Min Hwang.

**Resources:** Seong-Min Hwang.

**Supervision:** Jun-Mo Park.

**Writing – original draft:** Jun-Mo Park.

**Writing – review & editing:** Jun-Mo Park, Seong-Min Hwang.
